# Significance of clinical symptoms and red flags in early differential diagnosis of Parkinson’s disease and atypical Parkinsonian syndromes

**DOI:** 10.1007/s00702-023-02634-5

**Published:** 2023-04-12

**Authors:** Nils Schröter, Thilo van Eimeren, Joseph Classen, Johannes Levin, Christoph Redecker, Martin Wolz, Lars Tönges

**Affiliations:** 1 Department of Neurology and Clinical Neuroscience, Faculty of Medicine, Medical Center-University of Freiburg, University of Freiburg, Breisacher Str. 64, 79106 Freiburg, Germany; 2 Department of Neurology, Faculty of Medicine, University of Cologne, Cologne, Germany; 3 Department of Neurology, University of Leipzig, Leipzig, Germany; 4 Department of Neurology, University Hospital, Ludwig-Maximilians-Universität München, Munich, Germany; 5 German Center for Neurodegenerative Diseases, Site Munich, Munich, Germany; 6 Munich Cluster for Systems Neurology (SyNergy), Munich, Germany; 7 Department of Neurology, Lippe General Hospital, Lemgo, Germany; 8 Department of Neurology, Elblandklinikum Meißen, Meissen, Germany; 9 Department of Neurology, St. Josef-Hospital, Ruhr-University Bochum, Bochum, Germany

**Keywords:** Atypical Parkinson syndromes, PSP, CBS, DLB, MSA

## Abstract

The clinical presentation of Parkinson’s disease and atypical Parkinsonian syndromes is often heterogeneous. Additional diagnostic procedures including brain imaging and biomarker analyses can help to appreciate the various syndromes, but a precise clinical evaluation and differentiation is always necessary. To better assess the relevance of distinct clinical symptoms that arose within 1 year of disease manifestation and evaluate their indicative potential for an atypical Parkinsonian syndrome, we conducted a modified Delphi panel with seven movement disorder specialists. Five different topics with several clinical symptom items were discussed and consensus criteria were tested. This resulted in distinct symptom patterns for each atypical Parkinsonian syndrome showing the multitude of clinical involvement in each neurodegenerative disease. Strongly discriminating clinical signs were few and levels of indication were variable. A prospective validation of the assessments made is needed. This demonstrates that both clinical evaluation and elaborate additional diagnostic procedures are needed to achieve a high diagnostic standard.

## Introduction

The Parkinsonian syndromes are a pathologically and clinically heterogeneous group of neurodegenerative diseases that share a hypokinetic rigid syndrome due to nigrostriatal degeneration (Höglinger et al. 2017a). They can be clinically classified into Parkinson’s disease (PD), dementia with Lewy bodies (DLB) and multiple system atrophy (MSA), which together form the group of alpha-synucleinopathies. While the syndrome of progressive supranuclear palsy (PSP) is highly predictive of an underlying 4-repeat (4R) tauopathy, a corticobasal syndrome (CBS) shows great variability concerning the histopathological entity, including the 4-repeat (4R) tauopathies corticobasal degeneration, the CBS variant of PSP, but also the CBS variant of Alzheimer’s disease (Armstrong et al. 2013). Despite distinct phenotypes and rigorous international consensus criteria for each Parkinsonian syndrome, delineating these disorders poses a great clinical challenge in the first years as disease-defining symptoms have not yet developed or are interpreted in the context of alternative aetiologies (Litvan et al. 1997; Hohl et al. 2000; Josephs et al. 2006; Miki et al. 2019).

However, making an early correct diagnosis is of high relevance for the individual patient as the course of the diseases as well as the prognoses significantly differ. From a scientific view distinct disease modifying therapies targeting specific pathological hallmarks of the disorders with tau or alpha-synuclein pathology are under development. It is conceivable, that an early, targeted treatment initiation possibly could prevent or slow down future disability (Stamelou and Boxer 2015; Poewe et al. 2020).

The presence of disease-defining symptoms, which may be indicative for a specific Parkinson syndrome can be essential in making a diagnosis. Current diagnostic consensus criteria clearly support clinical decision making (Armstrong et al. 2013; Höglinger et al. 2017b; McKeith et al. 2017; Wenning et al. 2022). Due to the heterogeneous presentation of the diseases a definitive attribution of individual deficits to specific diseases is often difficult. In this study, therefore, an expert committee used the Delphi panel method to assess from a personal view how indicative the early presence of individual deficits within 1 year of disease manifestation are for the diagnosis of MSA, DLB, PSP or CBS, respectively.

## Methods

### Study design

A modified version of the Delphi study was conducted by seven movement disorder specialists with long standing experience in the differential diagnosis of neurodegenerative Parkinson disorders (JC, TvE, JL, CR, NS, LT, MW). This approach was chosen due to the broad and heterogeneous body of evidence for the significance of clinical symptoms and red flags within 1 year of disease manifestation in the differential diagnosis of neurodegenerative Parkinson’s syndromes. The strength of the method is the standardized implementation of repetitive rounds of questions, each with blinded evaluation and presentation of the interim results until the final assessment is reached. This prevents individual leaders from dominating the consensus-building process and reduces the risk of potential bias (Walker and Selfe 1996; Giannarou and Zervas 2014; Diamond et al. 2014). The study was conducted in three stages. (1) Within the framework of this study, the study participants identified symptoms/deficits according to previously defined categories (disease progression, ocular motor dysfunction, gait and posture, green flags, neuropsychology, oropharyngeal impairment) based on the current consensus guidelines (Armstrong et al. 2013; Postuma et al. 2015; Höglinger et al. 2017b; McKeith et al. 2017; Wenning et al. 2022). (2) Each deficit was rated in terms of how indicative it is of a specific atypical Parkinson syndrome (“Not Indicative”, “Possibly Indicative”, “Probably Indicative”). (3) Discussion of the results of the previous blinded rating was performed in a video conference, where results were expressed as absolute counts of the individual rating levels per deficit and disorder. Finally, a consensus was reached on how indicative each clinical feature is for the specific APS. Consensus was reached when all experts shared the same rating.

## Results

### Relevance of clinical features for differential diagnosis within 1 year of disease manifestation (see Table 1)

**Table 1:**
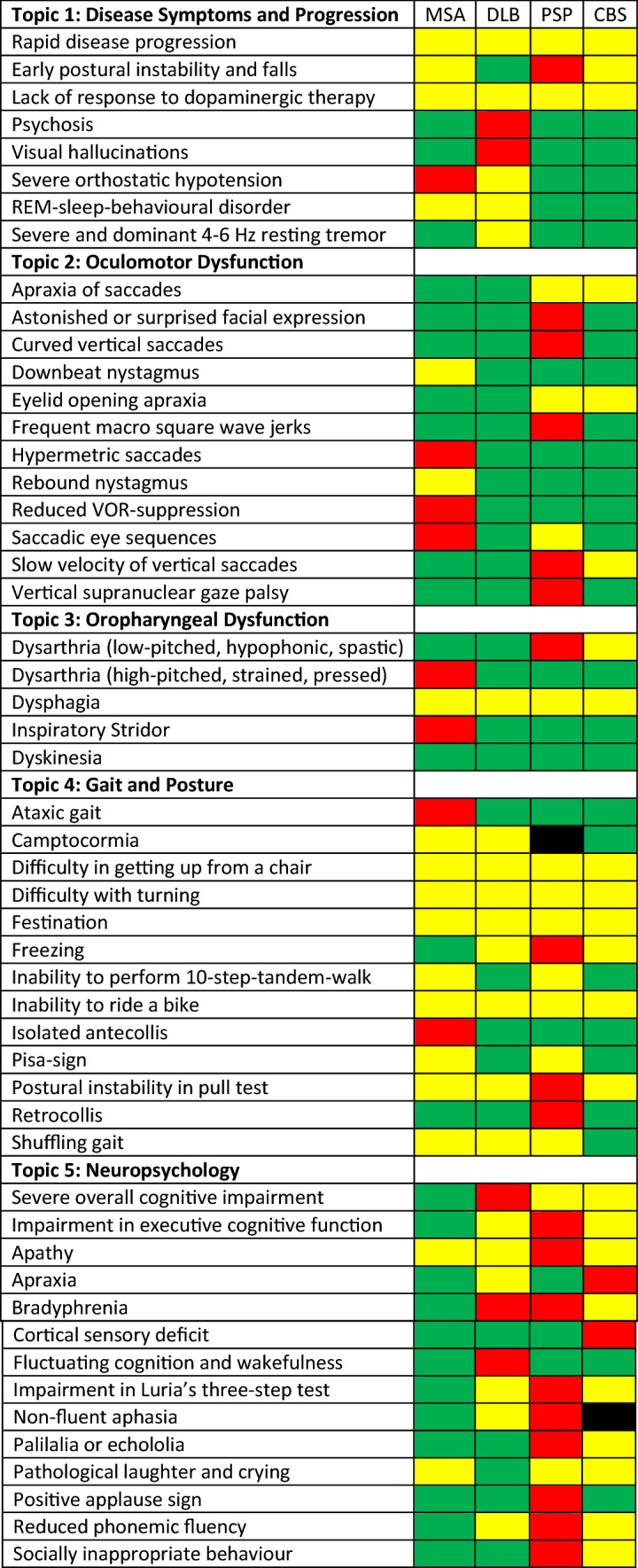
Consensus rating

Not indicative (Green), Possibly indicative (Yellow), Probably indicative (Red), No consensus (Black)

#### Topic 1: Disease-defining clinical symptoms and disease progression

Consensus rating: Early postural instability and falls are probably indicative for a PSP, possibly for MSA and CBS but not for DLB. While the lack of response to dopaminergic therapy and rapid disease progression are indicative for an APS, they are not indicative for a specific syndrome. Psychosis and visual hallucinations are probably indicative for DLB. Severe orthostatic hypotension is typically observed in MSA but can also be present in DLB but is not observed in the tauopathies. Similarly, the presence of REM sleep behavior disorder clearly argues against tauopathy. A severe and dominant 4–6 Hz resting tremor is not indicative for APS but can be observed in DLB.

#### Topic 2: Oculomotor dysfunction

Consensus rating: Apraxia of saccades and eyelid opening apraxia are indicative for tauopathies but not alpha-synucleinopathies. An astonished or surprised facial expression, curved vertical saccades, frequent macro square wave jerks and the presence of a vertical supranuclear gaze palsy are indicative for PSP. Hypermetric saccades and reduced VOR-suppression are probably, the presence of a downbeat or rebound nystagmus possibly indicative for MSA but not the other APS. Saccadic eye sequences are probably indicative for MSA and possibly indicative for PSP but not indicative for DLB and CBS.

#### Topic 3: Oropharyngeal dysfunction

Consensus rating: High-pitched, strained and pressed dysarthria and inspiratory stridor are probably indicative for MSA but not any other APS. Low-pitched, hypophonic and spastic dysarthria is probably indicative for PSP and possibly for CBS but not the synucleinopathies. Early dysphagia is indicative for any APS but does not allow further delineation. Dyskinesia are not indicative for any APS.

#### Topic 4: Gait and posture

Consensus rating: Difficulty in getting up from a chair or with turning as well as festination and the inability to ride a bike are indicative for APS but not assist in making a make specific diagnosis. Ataxic gate and isolated antecollis are probably indicative for MSA but not other APS. Camptocormia is possibly indicative for synucleinopathies but not CBS, no consensus was reached on camptocormia and PSP. Freezing is probably indicative for PSP and possibly for DLB and CBS but not MSA. The inability to perform a 10-step tandem walk and the presence of a Pisa sign are possibly indicative for MSA or PSP but not DLB and CBS. Postural instability is probably indicative for PSP but can be possibly indicative for any APS. Furthermore, the presence of a retrocollis is probably indicative for PSP but no other APS. A shuffling gait can be indicative for any APS but not CBS.

#### Topic 5: Neuropsychology

Consensus rating: Severe overall cognitive impairment is probably indicative for DLB, possibly for PSP and CBS but not MSA. Impaired executive function and observed in Luria’s three-step test is probably indicative for PSP, possibly for DLB and CBS but not MSA. Apathy is probably indicative for PSP but can also be seen in all other APS. A positive applause sign is probably indicative for PSP. Apraxia is probably indicative for CBS and possibly for DLB. Bradyphrenia is probably indicative for DLB and PSP, a cortical sensory deficit for CBS and fluctuating cognition and wakefulness for DLB. Non-fluent aphasia is probably indicative for PSP, no consensus was reached for CBS. Reduced phonemic fluency and socially inappropriate behavior are probably indicative for PSP.

## Discussion

In this modified Delphi study conducted by seven movement disorder specialists, we grouped clinical symptoms of PD and atypical parkinsonian syndromes into 5 clinical main topics including (1) disease-defining clinical symptoms and disease progression, (2) oculomotor dysfunction, (3) oropharyngeal dysfunction, (4) gait and posture and (5) neuropsychology. For each topic, several possible symptom presentations were discussed and its indicative strength for each disease was consented.

The progression of symptoms in atypical parkinsonian syndromes is typically different from PD because the progression of functional decline is often much more rapid. Early gait impairment and falls resulting in the need of assisting devices or wheelchair within 5 years of disease onset are common. Whereas a therapeutic response to dopaminergic therapy is typical in PD, in atypical parkinsonian syndromes the response may be poor or absent, and adverse effects can be more significant (McFarland and Hess 2017).

Cognitive impairment including hallucinations or psychosis can arise already in early disease stages (Weintraub et al. 2022; Bohnen et al. 2022). Patients with MSA only rarely manifest visual hallucinations (5%, compared to up of 75% in patients with Lewy body diseases) (Belvisi et al. 2018). Recurrent visual hallucinations (VH) not induced by drugs within 3 years of disease onset are an exclusion criterion for MSA in the latest diagnostic criteria (Wenning et al. 2022). It can be concluded that VH in the first years of disease onset is associated with a very low likelihood of MSA. Hallucinations and delusions are rarely observed in PSP (e.g., 0/24 PSP patients with a mean disease duration of approximately 3 years) (Belvisi et al. 2018). Predominant, otherwise unexplained visual hallucinations are an exclusion criterion in the latest PSP criteria (Höglinger et al. 2017b). It can be concluded that VH is associated with a very low likelihood of PSP. None of 37 autopsy-confirmed cases of CBD in a recent study showed VH. Hallucinations are mentioned as an exclusion criterion in the latest clinical criteria (Armstrong et al. 2013). It can be concluded that VH is associated with a very low likelihood of CBD.

Severe autonomic dysfunction may present rapidly with orthostatic hypotension (Wieling et al. 2022). In the most recent PSP diagnostic criteria, predominant, otherwise unexplained orthostatic hypotension (OH) is mentioned as an exclusion criterion (Höglinger et al. 2017b). A recent study in autopsy-confirmed cases corroborates this by finding OH to a very strong discriminating factor between PSP and synucleinopathies (PSP 0%; MSA, LBD 69%) (van Gerpen et al. 2019). Presence of severe, otherwise unexplained OH is associated with a very low likelihood of PSP. In 14 autopsy-confirmed cases of CBD, none had OH (Wenning et al. 1999). In the most recent CBD diagnostic criteria, prominent OH (severe dysautonomia in general) is mentioned as an exclusion criterion (Armstrong et al. 2013). OH presentation is not clearly different from PD, even though the latency tends to be shorter (Wenning et al. 1999). It is not mentioned as a criterion in the latest DLB criteria (McKeith et al. 2017). OH does not represent a non-supportive feature for DLB.

REM-Sleep-Behavioral Disorder is a rare phenomenon in 4R-tauopathies. Only 2 of 172 cases of PSG-confirmed and probable RBD cases that underwent autopsy, had PSP (Boeve et al. 2013). Notably, diffuse LBD can present as a CBS phenotype and then frequently shows a history of RBD (Kasanuki et al. 2018). Taken together, RBD can be seen as associated with a low likelihood of PSP or CBD, even though this feature is not mentioned as an exclusion criterion in the latest clinical criteria for PSP or CBD (Armstrong et al. 2013; Höglinger et al. 2017b).

Early accounts described absent or minimal resting tremor (RT) as a diagnostic hallmark of PSP (Golbe et al. 1988). However, RT is not mentioned as a feature reducing the likelihood of PSP in the newest criteria (Höglinger et al. 2017b). A recent retrospective study in more than 300 histopathologically confirmed cases of PSP specifically focused on the presence of tremor (Fujioka et al. 2016). 42% had a history of any form of tremor and 11% of resting tremor. However, the tremor severity in 96% of all patients was mild or less. Therefore, severe, dominant and typical RT could be seen as a feature associated with a very low likelihood of PSP. A recent review reports that up to 80% of MSA patients show tremor, most commonly in MSA-P. Resting tremor occurs in roughly one-third of patients. Only 10% show typical parkinsonian ‘‘pill-rolling’’ rest tremor. Classical “pill-rolling” rest tremor is mentioned in the latest MSA criteria as being infrequent and could therefore be seen as associated with a low likelihood of MSA (Kaindlstorfer et al. 2013; Wenning et al. 2022). RT is seen in approx. 20% of patients with pathologically confirmed CBD (Wenning et al. 1998; Kouri et al. 2011). Classical “pill-rolling” rest tremor is reported less frequently (1 of 14 patients) (Wenning et al. 1998). Literature is a bit sparse to derive a strong evidence-based statement. However, classic 4-Hz Parkinson disease resting tremor is mentioned as an exclusion criterion for CBD in the latest clinical diagnostic criteria (Armstrong et al. 2013). Tremor presentation is not clearly different from PD. It is not mentioned as a criterion in the latest DLB criteria (McKeith et al. 2017). RT does not represent a non-supportive feature for DLB.

In summary, postural instability and falls were found probably indicative for PSP but only possibly indicative for MSA and CBS. Lack of response to dopaminergic therapy and rapid disease progression were evaluated as indicative for an APS but not for a specific syndrome. Psychosis and visual hallucinations are probably indicative for DLB, whereas severe orthostatic hypotension is typically observed in MSA and not observed in the tauopathies. The presence of REM sleep behavior disorder is not seen in tauopathies. Severe and dominant 4–6 Hz resting tremor is not indicative for APS.

Oculomotor disturbances can occur early in the course of the disease in atypical Parkinson syndromes. These include eyelid opening and saccade apraxia, gaze palsy, nystagmus and various saccades disturbances caused by involvement of cerebellar structures as well as basal ganglia and areas of the brainstem. Due to the different impact of these structures in specific atypical Parkinson syndromes, the knowledge of oculomotor dysfunction can provide various clues to the underlying disease (Anderson and MacAskill 2013; Terao et al. 2016, 2017; Höglinger et al. 2017b; Kassavetis et al. 2022).

In the consensus rating, apraxia of saccades and eyelid opening apraxia were sees as indicative for tauopathies but not alpha-synucleinopathies. Clearly associated with PSP were astonished or surprised facial expression, curved vertical saccades, frequent macro square wave jerks and the presence of a vertical supranuclear gaze palsy. Hypermetric saccades and reduced VOR-suppression are probably indicative for MSA but not the other APS. Saccadic eye sequences are probably indicative for MSA and possibly indicative for PSP.

Unintelligible speech is often the first major disability experienced by patients with PSP. Dysarthria in PSP is typically mixed (Müller et al. 2001; Rusz et al. 2015) with predominant hypokinetic or spastic features (Kluin et al. 1993; Skodda et al. 2011). Dysarthria in PSP is dominated by increased dysfluency, decreased slow rate, inappropriate silences, deficits in vowel articulation and harsh voice quality(Rusz et al. 2015). Recent investigations further demonstrate that reduced pitch (f0 range) correlates with CSF pTau levels in some variants of PSP (Parjane et al. 2021). On the other hand, dysarthria in MSA patients typically exhibits ataxic features with high and fluctuating pitch, excess intensity variations, prolonged phonemes, vocal tremor and strained-strangled voice quality (Rusz et al. 2015).

Dysphagia is a common complaint of patients with PSP, MSA, and PD occurring much earlier and more severe in the course of PSP and MSA (Clark et al. 2020; Calandra-Buonaura et al. 2021). Dysphagic symptoms are usually already present when instrumentally assessed before becoming clinically evident (Higo et al. 2003; Suttrup et al. 2017). All stages of swallowing from oral preparation to oral, pharyngeal, and esophageal phases can be affected. Several findings suggest a common pathophysiological mechanism causing dysphagia in PD, MSA-P, and PSP, mainly related to the degeneration of cholinergic neurons of the pedunculopontine tegmental nucleus (Alfonsi et al. 2007; Schmeichel et al. 2008). The earlier occurrence and higher severity of dysphagia in MSA-P and PSP suggest additional dysfunction of brainstem central pattern generators of swallowing. MSA-P and PSP patients both frequently demonstrate a distinct opening deficit of the upper esophageal sphincter during the transit of the bolus from the pharynx to the esophagus (Alfonsi et al. 2007).

Laryngeal stridor is a frequent symptom of MSA (Cortelli et al. 2019). It has been included in the diagnostic criteria as additional feature for the diagnosis of possible MSA, showing high positive predictive value (Köllensperger et al. 2008; Ozawa et al. 2016). It has been suggested that its early occurrence might contribute to shorten survival.

Whereas dyskinesia in general are much more frequent in PD patients, dyskinesia was also described in 43% of pathologically confirmed cases of young onset MSA and 29% of these patients had an orofacial distribution of dyskinetic movements (Batla et al. 2018). Dyskinesia in PSP is rare and only found in single cases (Lang 2005). However, the occurrence of dyskinesia, respectively, dystonia of the oromandibular region and of the platysma (so called ‘risus sardonicus’), suggests MSA in the differential diagnosis (Jost et al. 2019).

In the consensus rating high-pitched, strained and pressed dysarthria and inspiratory stridor were seen as probably indicative for MSA but not any other APS. In contrast, low-pitched, hypophonic and spastic dysarthria are sees as probably indicative for PSP and possibly for CBS. Early dysphagia is indicative for any APS but does not allow further differentiation.

Abnormalities of posture, postural instability and gait difficulties and can occur at any stages of PD, although they are much more likely to start at later stages. The typical rigid akinetic gait disorder involves a slow gait with a short stride, a narrow foot often along with a stooped posture. In advanced stages, the arms are held in an adducted and flexed position. The feet are insufficiently elevated so that the gait is shuffling. The variability of the gait cycle from step to step increases. When patients are asked to walk faster, or even spontaneously, they increase the step frequency rather than the step length. Performing other tasks at the same time, such as walking while talking, worsens the gait. People with PD often find it easier to climb stairs than to walk on a flat surface. Many patients tend to lean forward when walking, which is associated with an increased step frequency, reduced stride length and a flexed trunk posture (Pirker and Katzenschlager 2017). This particular gait pattern in PD is called festination and carries the risk of forward falls. Changing direction when walking becomes more difficult with increasing axial bradykinesia. Patients turn en bloc with many small steps. Difficulty in gait initiation and freezing typically occur when turning or approaching obstacles or narrow passages such as doors. Freezing may or may not respond to antiparkinsonian medication. In PD patients with motor fluctuations, freezing may also occur during ON phases. Camptocormia refers to a forward bending of the trunk. The Pisa sign refers to a lateral bending of the trunk. Intact postural reflexes are necessary to mitigate the effects of a disturbance, e.g., due to an unexpected contact with an obstacle. Consequently, disturbed postural reflexes, as shown in the reaction to the pull test, are predictive of falls. Standing up from a sitting position is impaired, which is evident when patients are asked to stand up without using their arms.

In the case of postural instability, this is reflected in the fact that this symptom is considered a red flag when it occurs early in the course of the disease. Freezing is rather uncommon but can be present in variants such as, e.g., PSP with progressive gait freezing (PSP-PGF) (Höglinger et al. 2017b). Postural abnormality, such as antecollis, or retrocollis, may occur in Parkinson syndromes either with or without truncal postural abnormalities (Song et al. 2022).

In the consensus rating, difficulty in getting up from a chair or with turning as well as festination and the inability to ride a bike are seen as indicative for APS but do not differentiate single entities. Ataxic gate and isolated antecollis are probably indicative for MSA. Camptocormia is seen as possibly indicative for synucleinopathies whereas freezing is probably indicative for PSP. Postural instability and the presence of retrocollis are seen as probably indicative for PSP.

Neuropsychological deficits can be observed in all neurodegenerative parkinsonian syndromes. However, the spectrum of symptoms, the time of onset and the rate of progression can help to distinguish IPS from APS and to further delineate APS into definite diagnoses.

In patients newly diagnosed with PD, neuropsychological deficits are observed only in 10%, mostly consisting of impaired verbal memory and attention (Weintraub et al. 2015), rising up to a prevalence of 80% in the course of the disease (Aarsland et al. 2003). Early cognitive impairment within the first year after onset of the Parkinson syndrome and fluctuations of cognition and wakefulness are observed in LBD (Yamada et al. 2020). According to current consensus criteria, neuropsychological deficits are an exclusion feature for MSA (Wenning et al. 2022). However, frontotemporal deficits can be observed in advanced disease stages (O’Sullivan et al. 2008; Stankovic et al. 2014).

In PSP, neuropsychological deficits comprise executive dysfunction, apathy, or signs of disinhibition like positive applause sign that manifest early in the cause of the disease (Gerstenecker et al. 2013; Höglinger et al. 2017b; Fiorenzato et al. 2019). The diagnosis of CBD is mainly based on the prevalence of the cortical deficits apraxia, alien limb phenomenon and cortical sensory loss, already manifesting in early disease stages (Armstrong et al. 2013).

In the consensus, severe overall cognitive impairment is seen as probably indicative for DLB and possibly for PSP and CBS. Impaired executive function is probably indicative for PSP and possibly for DLB and CBS. A positive applause sign is probably indicative for PSP, whereas apraxia is probably indicative for CBS. Bradyphrenia is probably indicative for DLB and PSP and a cortical sensory deficit is probably indicative for CBS. Fluctuating cognition and wakefulness are probably indicative for DLB.

In summary, the modified Delphi panel draws distinct symptom patterns for each Parkinsonian syndrome and shows the multitude of clinical involvement in the neurodegenerative disease processes. However, discriminating clinical signs are few and vary with different levels of indication. To even better discriminate the disease entities, paraclinical information such as liquid or solid tissue biomarkers should be integrated into the diagnostic workup and a continuous re-evaluation of diagnostic guidelines should be performed.
